# Unraveling the temporal sequence of coronary atherosclerosis modification with lipid-lowering therapies through intravascular imaging: a narrative review

**DOI:** 10.3389/fcvm.2026.1737177

**Published:** 2026-01-22

**Authors:** Mingzhuang Sun, Zhenze Yu

**Affiliations:** Cardiac Department, Aerospace Center Hospital, Peking University Aerospace School of Clinical Medicine, Beijing, China

**Keywords:** atherosclerotic plaque regression, lipid-lowering therapy, PCSK9 inhibitors, plaque stabilization, statins

## Abstract

The management of ischemic heart disease has evolved from a narrow focus on low-density lipoprotein cholesterol (LDL-C) reduction to a comprehensive strategy targeting the regression and stabilization of coronary atherosclerotic plaque. Intravascular imaging modalities, including intravascular ultrasound (IVUS), optical coherence tomography (OCT), and near-infrared spectroscopy (NIRS), have been instrumental in characterizing the temporal sequence of plaque modification in response to lipid-lowering therapy. This review synthesizes evidence demonstrating that the effects on plaque are both time-dependent and agent-specific. Statins induce rapid plaque stabilization within weeks to months via mechanisms such asanti-inflammatory effects, fibrous cap thickening, and reduction of the lipid core. With prolonged treatment (months to years), statins promote plaque volume regression and facilitate a favorable shift in plaque composition towards a more stable, calcified phenotype. Non-statin agents further augment this regression. Ezetimibe, in combination with statins, provides synergistic LDL-C lowering and enhances plaque volume reduction. PCSK9 inhibitors, recognized as one of the most potent lipid-lowering agents currently available, have been shown in several studies to promote the regression of atherosclerotic plaques and reduce plaque volume. However, their effects on plaque composition—such as calcification, fibrous tissue, fibrofatty tissue, and necrotic core—remain controversial.

## 1 Introduction

Ischemic heart disease (IHD) remains a leading cause of global mortality and disability, pathologically rooted in the formation and progression of atherosclerotic plaque (1). Atherosclerosis, a chronic vascular pathology, is characterized by lipid deposition, inflammatory responses, and fibrous cap thinning, collectively leading to the formation of vulnerable plaques. The rupture or erosion of these vulnerable plaques exposes pro-thrombotic materials, activating the coagulation cascade and platelet aggregation, ultimately triggering occlusive thrombosis (atherothrombosis) and acute ischemic events (2, 3). The histological progression of human atherosclerotic lesions, as classified by the Committee on Vascular Lesions, progresses through six types (I–VI): it commences with lipoprotein accumulation and macrophage foam cell formation (Types I–II), advances to a lipid core (Types III–IV), and culminates in complex lesions comprising fibrous tissue, calcification, hemorrhage, or thrombosis (Types V–VI) (4). Traditional lipid-lowering therapy primarily focused on achieving target LDL-C levels. However, accumulating evidence indicates that major adverse cardiovascular events (MACE) are associated not only with the severity of coronary stenosis but, more critically, with the overall plaque burden and vulnerability features (5). For instance, thin-cap fibroatheroma (TCFA), characterized by a fibrous cap thickness <65 μm, a large lipid necrotic core, and macrophage infiltration, is a key precursor lesion of acute coronary syndrome (ACS) (6). The ROMICAT-II trial demonstrated that high-risk plaque features (positive remodeling, low-attenuation plaque, napkin-ring sign) detected by coronary computed tomography angiography (CCTA) in emergency department patients with chest pain independently predict ACS (7). Furthermore, CCTA-identified high-risk plaque features independently predict MACE (8).

Multiple studies utilizing IVUS, OCT, NIRS, and CCTA have corroborated that intensive lipid-lowering therapy can promote plaque regression. This review aims to systematically delineate the temporal effects of lipid-lowering therapy on coronary plaque volume, composition, and stability, and to explore the time-dependent efficacy of statin and non-statin medications in plaque management, thereby providing a theoretical foundation for clinical practice.

## 2 Assessment of plaque stability and regression

Accurate assessment of plaque characteristics is fundamental to investigating the temporal effects of lipid-lowering therapy. Currently, multiple imaging modalities are employed to evaluate plaque burden and composition (Table 1).

**Table 1:** Comparison of imaging modalities for coronary atherosclerotic plaque assessment.

Imaging modality	Principle	Key plaque characteristics assessed	Advantages	Limitations
Intravascular Ultrasound (IVUS)	Ultrasound	Percent atheroma volume (PAV) and total atheroma volume (TAV)	Evaluate plaque burden and vascular size	Limited tissue characterization; cannot accurately determine plaque composition.
Optical Coherence Tomography (OCT)	Near-infrared light	Fibrous cap thickness (FCT), lipid core, macrophage infiltration	High resolution; enables precise assessment of plaque vulnerability/instability	Weak penetration of infrared light; limitations in assessing total plaque burden and vessel dimensions
Coronary Computed Tomography Angiography (CCTA)	x-ray tomography	Qualitative and quantitative plaque analysis	Non-invasive; identifies coronary stenosis	Lower spatial resolution; heavy calcification can hinder interpretation
Near-Infrared Spectroscopy (NIRS)	Near-infrared spectroscopy	Detection and quantification of lipid content (Lipid Core Burden Index, LCBI)	Direct, quantitative measurement of plaque lipid content	No morphological/structural information
Positron Emission Tomography (PET)	Tracer metabolism (¹⁸F-NaF)	Identification of high-risk coronary plaques	Non-invasive assessment	Low spatial resolution; high cost; radiation exposure
Dual-Probe Molecular MRI	Targeted molecular probes	Plaque burden and inflammatory activity	First-in-class ability for multi-target evaluation in a single scan; novel method for *in vivo* quantification of biological features	In preclinical/early development phase; requires further clinical validation

### 2.1 Intravascular ultrasound (IVUS)

IVUS enables the quantitative assessment of plaque volume, utilizing key metrics such as percent atheroma volume (PAV) and total atheroma volume (TAV). It provides a precise evaluation of lumen and plaque characteristics, offering a comprehensive measure of plaque burden and overcoming the limitations of coronary angiography in lesion quantification (9). Beyond identifying high-risk lesions and optimizing revascularization strategies, IVUS is instrumental in assessing the efficacy of anti-atherosclerotic therapies, holding clinical value in coronary artery disease (CAD) research, therapeutic advancement, and prognosis prediction (10, 11). Advancements like radiofrequency IVUS analysis allow for real-time, accurate tissue characterization—differentiating fibrous, fibrofatty, calcified, and necrotic core components—with superior performance to traditional Fourier methods, providing an advanced tool for identifying vulnerable plaques and mitigating residual cardiovascular risk (12).

### 2.2 Optical coherence tomography (OCT)

OCT offers high resolution (10–20 micrometers), enabling precise measurement of fibrous cap thickness (FCT) and assessment of the lipid core and macrophage infiltration. This high-resolution intravascular imaging technique visualizes the microscopic structure of the coronary wall using infrared light, allowing for precise plaque characterization (13). Novel automated external elastic lamina (EEL) enhancement algorithms have improved the accuracy of plaque burden measurement by OCT (IVOCT), showing strong correlation with IVUS, particularly for fibroatheromas and mixed plaques, and enabling more accurate identification of high-risk lesions with plaque burden ≥70% (14). Regarding risk prediction, an OCT study revealed that a minimum FCT <80 μm and a representative FCT <188 μm are critical thresholds for predicting plaque rupture, outperforming the traditional autopsy-based standard of 65 μm (15). By revealing coronary plaque pathology and interventional details, OCT has become an indispensable tool for optimizing clinical decision and investigating vascular biology (13–16).

### 2.3 Coronary CT angiography (CCTA)

As a non-invasive imaging technique, CCTA can detect coronary stenosis and perform qualitative and quantitative plaque analysis, such as distinguishing lipid-rich from more fibrotic lesions (17). However, its resolution limits its ability to precisely identify specific features of vulnerable plaques (18). Nevertheless, validation data against intracoronary imaging and clinical outcomes support its potential for clinical application in vulnerable plaque identification and cardiovascular risk prediction. Particularly in low-to-intermediate risk patients, CCTA shows promise for monitoring plaque progression/regression and guiding personalized therapy, though its capability for assessing plaque subcomponents and its ultimate clinical utility require further investigation (19).

### 2.4 Emerging imaging technologies

Emerging imaging modalities offer unique perspectives on plaque assessment. NIRS directly detects plaque lipid content. Studies confirm the widespread presence of lipid-rich plaques in both culprit and non-culprit lesions of ACS patients, highlighting the diffuse nature of lipidic plaque and its association with clinical phenotypes, thereby offering a novel tool for assessing plaque biology and statin efficacy (20, 21). Intravascular optical imaging techniques, represented by NIRS and OCT, provide multi-dimensional information for high-risk plaque stratification and precision treatment by revealing microscopic structure, composition, and molecular features (22). Coronary Wall MRI can identify proximal coronary plaques, with measurements of lumen area and plaque burden highly correlating with IVUS, demonstrating potential for non-invasive assessment (23). Positron Emission Tomography (PET) transcends traditional anatomical imaging by targeting metabolic activities within plaques, such as inflammation and hypoxia. Specifically, ¹⁸F-sodium fluoride (¹⁸F-NaF) PET-CT non-invasively identifies high-risk coronary plaques, with higher uptake in culprit plaques of myocardial infarction patients, correlating with high-risk features like active calcification, showing unique value in risk stratification (24, 25). Dual-probe molecular MRI, utilizing different probes targeting elastin and macrophages, enables, for the first time, the simultaneous *in vivo* assessment of plaque burden and inflammatory activity within a single scan, offering a novel method for quantifying biological features at different stages of plaque progression (26). Furthermore, functionalized nanomaterial-based molecular imaging probes targeting key processes like inflammation and thrombogenesis, though facing clinical translation challenges, hold promise for breakthroughs in non-invasively assessing plaque instability and reshaping early diagnosis paradigms (27).

## 3 Plaque burden and cardiovascular outcomes

The accurate characterization of plaque burden and composition, as enabled by the imaging modalities described above, is critical because plaque burden is a key determinant of clinical outcomes. In a study of 581 patients with ACS or stable angina, IVUS revealed that small lumen area (≤4.0 mm^2^) and high plaque burden (≥70%) at coronary non-culprit lesions independently predicted long-term risk of adverse cardiovascular events in CAD patients (28). Another OCT study involving 1,474 patients undergoing PCI demonstrated that the presence of lipid-rich plaque (LRP) in the non-culprit region of the target vessel independently predicted an increased risk of future non-culprit lesion-related major adverse cardiac events (29).

## 4 Temporal effects of statin therapy

### 4.1 Early effects (weeks to months): anti-inflammatory and rapid plaque stabilization

Statinsexert rapid plaque-stabilizing effects within weeks through both lipid-lowering and non-lipid (pleiotropic) pathways (Table 2).

**Table 2:** Chronological evolution of coronary atherosclerotic plaque under intensive lipid-lowering therapy.

Phase	Time window	Plaque evolution
Rapid Stabilization	Weeks to 3 months	Rapid fibrous cap thickening (31), swift reduction of the lipid core (33), and marked decrease in plaque inflammation (30). Plaque rupture risk declines.
Early Regression	3 to 12 months	Definite initiation of plaque volume reduction (35), necrotic core decrease (40), and increase in fibrous tissue (36). Positive vascular remodeling emerges.
Advanced Remodeling & Regression	>12 months (1–2 years)	Plaque volume regression (47, 71), a calcification shift towards a stable phenotype (71).

Anti-inflammatory Action: Statinsexert anti-inflammatory effects via non-lipid pathways. A study showed that atorvastatin 80 mg reduced arterial wall FDG uptake (TBR) by 14.42% (*P* < 0.001) after 12 weeks, with a effect observed as early as 4 weeks (12.5% TBR reduction), independent of lipid changes (30).

Fibrous Cap Thickening: Statin therapyincreases FCT, a key indicator of plaque stability. The ESCORT study demonstrated that early initiation of pitavastatin (4 mg/d) in ACS patients increased FCT by 20 μm within 3 weeks, whereas the delayed treatment group exhibited FCT thinning, between baseline and 36-week follow-up, fibrous-cap thickness increased comparably in the 2 groups (31). A meta-analysis confirmed that statin therapy increased mean FCT by 58.79 μm (*P* < 0.001), an effect superior to placebo (32).

Lipid Core Reduction: In the YELLOW trial, rosuvastatin 40 mg/d for 7 weeks reduced the lipid core burden index (LCBI) in obstructive lesions (median reduction of 149.1), indicating that short-term intensive statin therapy depletes plaque lipid content (33).

Notably, early effects may vary among different statins. One study comparing ACS patients undergoing emergency PCI found that after 2–3 weeks of treatment, the pitavastatin group showed reductions in plaque volume index and fibrofatty volume index, while the atorvastatin group did not, potentially due to differences in pharmacokinetics and plaque response (34).

### 4.2 Medium- to long-term effects (months to years): plaque regression and composition change

#### 4.2.1 Statin therapy

Prolonged statin therapy may not only further stabilizes plaques but also promotes plaque volume regression and favorable compositional changes, ultimately translating into clinical benefit.

Plaque Volume Reduction: Plaque volume reduction can be observed as early as 6 months in some studies. Multiple studies have confirmed that statin treatment for 6 to 12 months can increase fibrous composition, reduce lipids and the volume of necrotic cores (35–39). The STABLE study showed that one year of rosuvastatin therapy reduced necrotic core volume and the incidence of thin-cap fibroatheroma (40). The EASY-FIT RCT demonstrated that atorvastatin 20 mg/d was more than 5 mg/d in thickening the fibrous cap and lowering LDL-C after 12 months (41). At 12–13 months, the stability of plaque components has improved, manifested as an increase in the hyperechoic index of the plaque (42), thickening of the non-culprit lesion fibrous cap, reduction of macrophage infiltration, and morphological reversal of nearly 70% of thin-cap fibrous atherosclerotic plaques (43). By 18 months, both moderate-intensity and high-intensity treatments could delay or regress the progression of coronary atherosclerosis (44–46). The ASTEROID trial first provided evidence that intensive rosuvastatin 40 mg therapy for 24 months could induced plaque regression (mean PAV reduction of 0.98%) (47). The SATURN study showed that ACS patients receiving 24 months of high-intensity statin therapy had more pronounced plaque regression (48).

Calcification Shift: Statin therapy promotes the transformation of non-calcified plaque into calcified plaque. Although this increases the overall calcification burden, it enhances plaque stability. The PARADIGM study showed that statin users had increased annual progression of calcified plaque and attenuated progression of non-calcified plaque (49). A prospective study showed that after 8 months of therapy, both intensive and moderate lipid-lowering reduced fibrofatty plaque volume while increasing the calcified component (38). A study with a mean follow-up of 6.2 years found that statin use was independently associated with increased calcified plaque progression and decreased non-calcified plaque progression (50).

Dose-Dependent Effect: The plaque-regressive effect of statins is clearly dose-dependent. A 6-month study showed that atorvastatin 40 mg/d reduced plaque volume more than 10 mg/d and limited necrotic core expansion (51). Rosuvastatin 10 mg/d for one year reduced necrotic core volume, whereas simvastatin 20 mg/d did not (52). An observational study over >2 years found that achieving LDL-C < 70 mg/dL slowed plaque progression (53).

Effect in Special Populations: Plaque continues to progress more readily in diabetic patients even when LDL-C targets are met (54). CKD patients exhibit greater plaque burden, with higher proportions of necrotic core and calcium (55).

#### 4.2.2 Additive effects of non-statin therapies ezetimibe: synergistic lipid-lowering and plaque regression

Ezetimibe, which inhibits intestinal cholesterol absorption, provides synergistic LDL-C lowering when combined with statins. Clinical endpoint trials such as IMPROVE-IT confirm that this combination reduces major cardiovascular events, supporting the benefit of deeper LDL-C reduction (56).

Across multiple intravascular imaging studies spanning 6–12 months, the addition of ezetimibe to statin therapy promotes plaque volume regression compared to statin monotherapy (57–60). For instance, the PRECISE-IVUS trial demonstrated reductions in percent atheroma volume (PAV) and a higher proportion of patients achieving plaque regression with combination therapy (59). Although combination therapy intensifies lipid-lowering, changes in plaque composition may not be (61), and plaque color improvement may be comparable to statin monotherapy (62).

However, one prospective study in ACS patients found that despite achieving lower LDL-C, the combination of pitavastatin and ezetimibe did not result in greater regression of total atheroma volume or lipid plaque compared to statin alone (63).

#### 4.2.3 PCSK9 inhibitors: potent lipid-lowering and rapid plaque regression

PCSK9 inhibitors can reduce LDL-C in high-risk patients and are associated with a further reduction in atherosclerotic cardiovascular events (64). PCSK9 inhibitors achieve profound LDL-C reductions (55%–72%) and further reduce atherosclerotic cardiovascular events in high-risk patients, as evidenced by outcome trials like FOURIER and ODYSSEY OUTCOMES (65, 66).

Collective evidence from multiple imaging studies indicates that PCSK9 inhibitors exert a clear time-dependent and dose-response effect on coronary plaque regression. Short-term studies (12–36 weeks) often fail to demonstrate significant changes in plaque volume (67, 68). In contrast, long-term treatment demonstrates efficacy in promoting plaque regression. A ∼12-month study showed that adding a PCSK9 inhibitor to statins resulted in greater LDL-C reduction (between-group difference 46.4%) and more regression of non-culprit plaques, including reductions in PAV and maxLCBI4mm (69), the GLAGOV trial (76 weeks) showed that evolocumab induced a 0.95% regression in percent atheroma volume (PAV) and increased the proportion of patients achieving plaque regression (70). The PACMAN-AMI trial (52 weeks) further confirmed that alirocumab not only reduced PAV and lipid burden but also increased fibrous cap thickness, with nearly one-third of patients achieving increased fibrous c(simultaneous improvement in volume, lipid content, and cap thickness), which was associated with a reduced risk of major adverse cardiovascular events (71, 72).

Radiofrequency analysis of the GLAGOV study indicated that while adding evolocumab to statins further reduced LDL-C and promoted TAV regression, there were no differences in the changes of calcified, fibrous, fibrofatty, and necrotic core plaque components between groups (73). Another study showed that adding alirocumab to high-intensity statins for 78 weeks reduced total coronary plaque burden by 4.6% in patients with familial hypercholesterolemia, with greater regression observed in patients with higher baseline plaque burden and a greater proportion of unstable core (74). In a subsequent analysis of the PACMAN-AMI trial (75), which included 245 patients and a total of 591 high-risk non-culprit lesions with baseline plaque burden ≥ 40%, a greater reduction in percent atheroma volume (PAV) was observed (−4.86% vs. −2.78%). The reduction in PAV was particularly pronounced at the site of minimal lumen area (MLA) (−10.14% vs. −6.70%). These findings contrast with those from earlier short-term lipid-lowering therapy (12 weeks) (68). The HUYGENS study showed that evolocumab for 52 weeks increased minimum FCT, decreased maximum lipid arc, and reduced plaque volume (76) (Table 3).

**Table 3:** Key randomized controlled trials of lipid-lowering therapy: imaging follow-up and plaque outcomes.

Study	Intervention	Endpoints	Follow-up	Key finding	Year	Patient	Sample size	Assessment method
Toi et al. (34)	Pitavastatin 2 mg/Atorvastatin 10 mg	PVI, FFVI	2–3 weeks	PVI and FFVI reduced in the pitavastatin group. (*P* < 0.05) No significant changes observed in the atorvastatin group.	2009	ACS-PCI patients	160	VH-IVUS
YELLOW (33)	Rosuvastatin 40 mg/d/Standard care	LCBI4 mm max	7 weeks	LCBI4 mm max significantly reduced. (−149.1 [−210.9 to −42.9] vs. 2.4 [−36.1 to 44.7]; *P* = 0.01)	2013	Patients with multivessel CAD	87	FFR, NIRS, IVUS
Tawakol et al. (30)	Atorvastatin 80 mg/10 mg	TBR	4, and 12 weeks	inflammation (TBR) in the index vessel was significantly reduced from baseline with atorvastatin 80 mg (% reduction [95% confidence interval]: 14.42% [8.7% to 19.8%]; *P* < 0.001)	2013	Patients with risk factors or established ASCVD, not on high-dose statins	67	FDG-PET/CT
ESCORT (31)	Pitavastatin 4 mg/d (Early/Delated initiation)	FCT	3-week, and 36-week	Between baseline and 3-week follow-up, FCT increased in the early statin group (140 μe [interquartile range (IQR):120 to 170 μ[] to 160 μ [IQR: 130 to 190 μ[]; *P* = 0.017), but decreased in the late statin group (135 μ. [IQR: 110 to 183 μ[] to 130 μ [IQR: 108 to 160 μ(); *P* = 0.020]. Between baseline and 36-week follow-up, fibrous-cap thickness increased comparably in the 2 groups.	2017	ACS patients	53	OCT
ESTABLISH (35)	Atorvastatin 20 mg/d/Conventional diet or cholesterol inhibitor	PV	6 months	Plaque volume was significantly reduced in the atorvastatin group (13.1 +/− 12.8% decrease) compared with the control group (8.7 +/− 14.9% increase; *P* < 0.0001)	2004	ACS-PCI patients	48	IVUS
Masuda et al. (58)	Rosuvastatin 5 mg/d + Ezetimibe 10 mg/d/Rosuvastatin 5 mg/d	PV	6 months	PV appeared to decrease more effectively in the combination group compared with the monotherapy group (−13.2% versus −3.1%, respectively, *P* = 0.050).	2015	PCI patients	51	IVUS
JAPAN-ACS (39)	Atorvastatin 20 mg/Pitavastatin 2 mg	PV	8–12 months	The mean percentage change in PV was −16.9 +/− 13.9% and −18.1 +/− 14.2% (*P* = 0.5) in the pitavastatin and atorvastatin groups	2009	ACS patients	252	IVUS
STABLE (40)	Rosuvastatin 40 mg/10 mg	VH intravascular ultrasound-defined fibroatheroma-containing index lesion	12 months	NC volume within the target segment significantly decreased from 21.3 ± 6.8% to 18.0 ± 7.5% during 1-year follow-up, whereas the percent fibrofatty volume increased (11.7 ± 5.8% vs. 14.8 ± 9.3%; all *P* < 0.001).	2016	Patients with coronary atherosclerosis	225	VH-IVUS
EASY-FIT (41)	Atorvastatin 20 mg/5 mg	FCT	12 months	The increase in ﬁbrous cap thickness was signiﬁcantly greater with 20 mg/day compared with 5 mg/day of atorvastatin (69% vs. 17%; *P* < 0.001).	2014	Patients with unstable angina and untreated dyslipidemia	70	OCT
OCTIVUS (60)	Atorvastatin 80 mg + Ezetimibe/Atorvastatin 80 mg	NC,PAV,TAV	12 months	ezetimibe group 24.9 (11.9, 51.3) mm^3^ to 24.9 (15.3, 54.5) mm^3^, *P* = 0.76, placebo group 29.4 (16.3, 78.5) mm^3^ to 32.0 (16.0, 88.7) mm^3^, *P* = 0.30, (*P* = 0.35 between groups). PAV was reduced in the ezetimibe group only (40.1 ± 8.6% to 39.2 ± 9.0%, *P* = 0.036)	2016	Patients with STEMI	87	IVUS
PACMAN-AMI (71)	Alirocumab vs Placebo (2 groups both on 20 mg of rosuvastatin)	PAV, TAV,FCT, LCBI4 mm max	52 weeks	mean change in PAV was −2.13% with alirocumab vs −0.92% with placebo [difference, −1.21% (95% CI, −1.78% to −0.65%), *P* < .001]. Mean change in maximum lipid core burden index within 4 mm was −79.42 with alirocumab vs. −37.60 with placebo [difference, −41.24 (95% CI, −70.71 to −11.77); *P* = .006]. Mean change in minimal FCT was 62.67 μm with alirocumab vs 33.19 μm with placebo [difference, 29.65 μm (95% CI, 11.75–47.55); *P* = .001].	2022	ACS patients	265	IVUS, NIRS, OCT
GLAGOV (70)	Statin + Evolocumab/Statin + Placebo	PAV, TAV and percentage of patients demonstrating plaque regression	76 weeks	Evolocumab induced plaque regression in a greater percentage of patients than placebo [64.3% vs 47.3%; difference, 17.0% [95% CI, 10.4% to 23.6%]; *P* < .001 for PAV and 61.5% vs 48.9%; difference, 12.5% [95% CI, 5.9% to 19.2%]; *P* < .001 for TAV].	2016	CAD patients on statins	968	IVUS
ASTEROID (47)	Rosuvastatin 40 mg	PAV, TAV	24 months	The mean (SD) change in PAV for the entire vessel was −0.98% (3.15%), with a median of −0.79% (97.5% CI, −1.21% to −0.53%) (*P* < .001 vs baseline).	2006	Patients undergoing coronary angiography	349	IVUS

PVI, plaque volume index; FFVI, fibro-fatty volume index; LCBI4 mm max, lipid-core burden index at the 4-mm maximal segment; TBR, target-to-background ratio; FCT, fibrous-cap thickness; PV, plaque volume; PAV, percent atheroma volume; TAV, total atheroma volume; VH, virtual histology; NC, Percent necrotic core.

## 5 Individualized treatment strategies and clinical pathways

Plaque management-guided lipid-lowering therapy should adhere to individualized principles, fully considering patient risk stratification, clinical characteristics, and treatment response to maximize clinical benefit.

### 5.1 Risk stratification-guided lipid goals and drug selection

The intensity of lipid-lowering must be strictly matched to the patient's risk stratum. For very high-risk/extremely high-risk patients (e.g., those with ACS or multivessel disease), international guidelines generally recommend stricter LDL-C targets [e.g., <1.4 mmol/L (∼55 mg/dL) with a >50% reduction from baseline]. Statins remain the cornerstone, but early combination with non-statin agents (ezetimibe or PCSK9 inhibitors) is increasingly emphasized to achieve deep lipid-lowering (77). Studies like PACMAN-AMI (71) confirm that early initiation of statin combined with a PCSK9 inhibitor in ACS patients and potently promotes plaque regression and stabilization.

### 5.2 Dynamic management strategy based on treatment timeline

Current guidelines do not routinely recommend repeated imaging surveillance to guide lipid-lowering therapy adjustment. While imaging response is associated with plaque improvement, there is still a lack of high-level evidence supporting its ability to independently improve hard clinical endpoints or its cost-ness. Therefore, this strategy should be regarded as an exploratory framework awaiting validation by prospective studies, rather than an established clinical standard.

## 6 Summary and future perspectives

Intensive lipid-lowering therapy is associated with a clear, time-dependent sequence of changes in coronary plaques: the early phase (weeks to months) is characterized by rapid stabilizes plaques primarily through anti-inflammatory effects, FCT thickening, and lipid core reduction; the medium- to long-term phase (months to years) is further associated with plaque volume regression, favorable compositional transformation (increased calcification), and ultimate clinical benefit. Statins, ezetimibe, and PCSK9 inhibitors synergistically contribute to this process through distinct mechanisms, with PCSK9 inhibitors demonstrating the most potent plaque-regressive efficacy.

Early-stage lipid-lowering therapy (over weeks to months) are dominated by rapid stabilization, manifested as reduced inflammation and fibrous cap thickening, whereas medium- to long-term outcomes (over months to years) gradually involve plaque volume regression and a shift toward calcification (78). This “time-axis” perspective moves beyond the simplistic question of “whether it works” and addresses the clinically relevant issue of “when specific changes occur”, thereby providing a direct theoretical foundation for implementing time-sensitive, individualized treatment strategies. In contrast to recent reviews that have predominantly focused on specific populations (e.g., patients with diabetes) (79), we systematically examines the temporal efficacy of lipid-lowering therapy in a broad atherosclerotic population.

Different imaging modalities (OCT/IVUS/NIRS/PET/CCTA) vary in their temporal resolution and underlying principles, making direct comparisons of timing across techniques unreliable.While intravascular imaging techniques such as optical coherence tomography (OCT) and intravascular ultrasound (IVUS) offer high resolution, their measurements are subject to inter-observer and intra-luminal variability. Therefore, micron-scale or percent-level changes should be interpreted cautiously within the context of long-term, serial follow-up. More importantly, these morphological improvements—though serving as surrogate endpoints that reflect favorable shifts in plaque biology—do not yet have fully established direct links to individual hard clinical endpoints such as myocardial infarction or cardiovascular death. Clinical decision-making should continue to be guided primarily by outcome evidence from large randomized controlled trials.

Looking forward, therapeutic strategies may extend beyond lipid-centric approaches. For instance, novel anti-inflammatory therapies represent a promising avenue for inducing plaque regression. A prospective study demonstrated that in post-myocardial infarction patients with residual inflammation, MEDI6570 potently and dose-dependently inhibited its target (LOX-1) and downregulated the downstream inflammatory pathway (IL-6). However, this effect did not translate into short-term (32-week) plaque volume reduction (80), underscoring the need to explore optimal timing, patient selection, and combination strategies.

Future research directions include: (1) exploring more precise plaque risk assessment systems integrating imaging, biomarkers, and genetic information; (2) optimizing lipid-lowering strategies for different populations, especially special groups like diabetic and CKD patients; (3) developing novel lipid-lowering drugs to comprehensively target all pro-atherogenic lipid components, including LDL-C, lipoprotein(a) [Lp(a)], and remnant cholesterol; and (4) leveraging AI-based plaque analysis technologies for more precise dynamic plaque monitoring.

Panvascular diseases share atherosclerosis as a common pathological basis. Modern lipid-lowering therapy must transcend the simplistic goal of numerical target achievement and shift towards comprehensive management focused on plaque regression and vascular repair. By matching lipid-lowering intensity to risk stratification, emphasizing early intervention and long-term maintenance, adopting multi-target intervention strategies, and following a clinical pathway of “Screen-Regress-Monitor-Maintain” guided by advanced imaging to assess plaque burden, a reduction in cardiovascular events can ultimately be achieved.

## 7 Conclusions

While intravascular imaging defines a clear timeline of plaque benefit (stabilization to regression), this imaging evidence currently explains how treatment works rather than dictates how to treat; routine clinical practice must still be guided by outcomes from large trials.
